# 靶向治疗背景下非小细胞肺癌传统化疗的地位与发展

**DOI:** 10.3779/j.issn.1009-3419.2015.09.10

**Published:** 2015-09-20

**Authors:** 国伟 张, 慧娟 王, 米娜 张, 鹏 李, 智勇 马

**Affiliations:** 450003 郑州，郑州大学附属肿瘤医院（河南省肿瘤医院）内科 Department of Internal Medicine, The Affiliated Cancer Hospital of Zhengzhou University, Henan Cancer Hospital, Zhengzhou 450003, China

**Keywords:** 肺肿瘤, 化疗, 靶向治疗, Lung neoplasms, Chemotherpy, Targeted therapy

## Abstract

近年来随着非小细胞肺癌靶向治疗快速发展，传统化疗获得的关注越来越少，而如何定位并合理地应用传统化疗药物，使患者从中得到最大的获益，在当前仍是不可忽视的命题。为此，通过对相关文献的回顾和分析，本文指出无论有无驱动基因突变的患者，传统化疗仍有许多不可替代之处，并且演变出了一些新的治疗模式，进一步提高了患者的生存获益，从而明确了靶向治疗背景下传统化疗的地位。同时通过对化疗疗效预测分子标志物研究和发展的阐述，指出未来传统化疗必将“靶向化”的发展方向。

## Abstract (en)

In recent years, along with rapid development of targeted therapy in non-small cell lung cancer, traditional chemotherapy get less and less attention. Yet it still can not be ignored in the current that how to locate and use traditional chemotherapy so patients could derive maximum benefit. For this purpose, through the literature review and analysis, we point out there are still many traditional chemotherapy irreplaceable places whatever patients' driver gene status. And there are some new treatment modalities of traditional chemotherapy which have been developed to further improve patients' survival. At the same time, through exposition of predictive bio-markers development in chemotherapy, we pointed out that the future of traditional chemotherapy must be part of "targeted therapy".

近十余年来，非小细胞肺癌（non-small cell lung cancer, NSCLC）的治疗获得了长足的发展，多种靶向药物已成熟地应用于临床，显著延长了患者的生存。在这样的背景下，NSCLC传统的含铂双联化疗逐渐被边缘化。化疗是否将逐渐被靶向治疗取代？当前化疗在NSCLC治疗中的地位及其未来的发展前景究竟如何？本文试图就目前NSCLC传统化疗的地位及其发展趋势做出分析和阐述。

## 1 传统化疗在驱动基因突变NSCLC治疗中的作用和地位

在无驱动基因突变的NSCLC中，传统化疗仍是标准治疗自不待言。而在存在驱动基因突变的NSCLC中，相应的小分子酪氨酸激酶抑制剂是近年来NSCLC治疗领域获得的最重要成就。即便如此，在驱动基因突变NSCLC中，化疗仍有其重要地位。由于间变性淋巴瘤激酶（anaplastic lymphoma kinase, ALK）抑制剂目前问世时间尚短，积累的循证学证据较少，且目前发展轨迹与表皮生长因子酪氨酸激酶抑制剂（epidermal growth factor receptor tyrosine kinase inhibitor, EGFR-TKI）类似。因此我们以下主要以EGFR突变的NSCLC展开分析。

### 1.1 EGFR-TKI尚无法取代辅助化疗

由于辅助治疗的临床研究周期较长，早期EGFR-TKI临床研究开展时*EGFR*突变的疗效预测意义尚未确定，因此并未把入组人群局限在突变患者。如2002年开展的BR19是最早关于EGFR-TKI术后辅助治疗的Ⅲ期临床试验^[1]^，于2005年4月提前终止，共入组503例Ⅰb期-Ⅲa期泛人群NSCLC术后患者，术后随机接受最长2年的吉非替尼辅助治疗或安慰剂（2003年以后两组患者也随机进行了术后辅助化疗）。结果显示吉非替尼辅助治疗无病生存期（disease-free survival, DFS）和总生存期（overall survival, OS）均无获益，甚至增加了死亡风险。该研究虽然失败，但由于存在太多不利因素：如研究提前终止，Ⅰb期患者过多（49%），入组人群为*EGFR*突变率很低的高加索裔非选择性人群，吉非替尼中位辅助治疗时间过短（4.8个月）。因此多认为该研究不足以作为否定术后辅助EGFR-TKI治疗的证据。

2014年美国临床肿瘤学会（American Society of Clinical Oncology, ASCO）年会报告了两项关于EGFR-TKI术后辅助治疗的Ⅲ期临床试验：RADIANT^[2]^和SELECT^[3]^。RADIANT入组了973例EGFR免疫组化或FISH阳性的Ⅰb期-Ⅲa期完全切除术后的NSCLC患者，允许接受过辅助化疗180天以内的患者入组，2:1随机接受最多2年的厄洛替尼或安慰剂，结果显示DFS和OS差异均无统计学意义。亚组分析显示，*EGFR*突变者接受厄洛替尼辅助治疗中位DFS较安慰剂组延长17.9个月，但根据试验设计采用的*Hierarchical*检验未达统计学差异，OS也未见延长。SELECT是一项单臂的多中心Ⅱ期临床研究，入组100例Ia-Ⅲa期*EGFR*突变阳性患者，在术后和标准辅助化疗后接受最长2年的厄洛替尼辅助治疗，以2年DFS率为主要终点。结果显示2年DFS率为89%，超过历史对照的76%，存在统计学意义（*P*=0.004, 7）。截至报告时为止，中位DFS尚未达到。

由以上研究可知，NSCLC敏感型突变患者术后接受TKI辅助治疗可能延长DFS，但目前数据或无统计学意义，或为Ⅱ期单臂研究，证据级别有限。况且RADIANT和SELECT研究均未敢完全放弃标准的辅助化疗，EGFR-TKI若要取代术后辅助化疗必须要有与辅助化疗头对头的随机对照临床试验。因此，以目前的循证医学证据，即便是EGFR敏感型突变患者，含铂双联化疗也仍是标准的术后辅助治疗，其地位短期之内不可取代。

### 1.2 EGFR-TKI尚无法取代局部晚期NSCLC的化疗

同辅助治疗一样，在*EGFR*突变的意义尚未明确时，已经有临床试验开始探索EGFR-TKI在局部晚期与放疗的联合。2002年开始的Ⅱ期临床试验CALGB 30106^[4]^，入组63例Ⅲ期NSCLC患者，先给予两个周期的吉非替尼联合诱导化疗，随后按照患者的风险分层接受不同的治疗，高风险者（体重下降≥5%和/或体能状态评分=2分）接受吉非替尼同步放疗，低风险者（体重下降 < 5%且体能状态评分≤1分）接受同步化放疗加吉非替尼，化放疗结束后TKI持续应用至病情进展。结果显示高风险者OS反而长于低风险者，而在EGFR突变患者中，高风险者（*n*=5）OS同样长于低风险者（*n*=6），分别为28.4个月和7.2个月。低风险者同步化放疗联合TKI反而得到了更差的结果，这使得该研究于2005年提前终止。另一项Ⅲ期临床试验SWOG S0023在Ⅲ期患者同步化放疗（依托泊苷/顺铂）后给予3个周期的多西他赛单药化疗^[5]^，之后随机接受吉非替尼或安慰剂至病情进展，结果显示吉非替尼组OS甚至差于安慰剂组（23个月*vs* 35个月，*P*=0.013），因此该试验也提前终止了。

以上结果提示局部晚期同步化放疗联合EGFR-TKI是不可取的，甚至对患者是可能有害的，而在*EGFR*突变患者中TKI单独与放疗联合可能是可行的治疗模式，但需要有高级别循证医学证据的支持。目前在临床实践中，EGFR-TKI在局部晚期NSCLC的治疗中是没有任何地位的，传统的联合化放疗仍是其标准治疗。

### 1.3 传统化疗在*EGFR*突变晚期NSCLC中仍有重要地位

在*EGFR*敏感型突变的晚期NSCLC中，EGFR-TKI无疑是最重要的治疗药物，他已经可以取代含铂双联化疗作为标准的一线治疗。但是，如果以全局的观念看待晚期*EGFR*突变患者的治疗，传统化疗的存在仍有重要的意义。此观点主要建立在临床试验OPTIMAL上^[6]^，这是我国学者进行的*EGFR*敏感突变晚期NSCLC患者中一线厄洛替尼对比化疗的经典临床试验，按患者的一线及后续治疗进行了亚组分析，按患者的全局治疗将患者分为仅接受过厄洛替尼、仅接受过化疗、既接受过厄洛替尼也接受过化疗三个亚组，结果显示既接受过厄洛替尼也接受过化疗的患者中位OS最长达30.4个月，仅接受过TKI的患者次之，达20.6个月，差异存在统计学意义（*P*=0.000, 1）。这说明在晚期EGFR突变患者的治疗全程中化疗贡献了约1/3的OS，一线EGFR-TKI治疗失败后，传统化疗仍是重要的治疗手段。

综上所述，即便在存在驱动基因突变的患者中，传统化疗在辅助化疗及局部晚期NSCLC的治疗中的地位仍不可取代，即便是在晚期患者中，化疗的合理应用也可为患者的生存获益做出不可或缺的贡献。

## 2 NSCLC化疗应用的新模式

随着众多新药物不断应用于临床，既然传统化疗远不能退出历史舞台，研究者们开始为其寻求变通，寻求新的应用模式。其中最为常见的就是化疗与靶向药物的联合，以及维持化疗的出现。

### 2.1 化疗与靶向药物的联合

自从靶向药物出现，学者们就开始探索其与传统化疗药物的联合，期待两类有效药物的组合能给患者带来更长的生存。EGFR-TKI问世之初就曾在高加索裔泛人群中进行过与化疗联用的探索，但遗憾的是均以失败告终。2013年，FASTACT-2研究使用一线化疗周期内厄洛替尼插入治疗的模式与一线化疗加安慰剂对比^[7]^，该研究入组人群为亚裔非选择人群，结果显示在客观缓解率（objective response rate, ORR）、无进展生存期（progression-free survival, PFS）、OS均有显著获益，EGFR-TKI与化疗联合的探索至此才得到了部分肯定。但事实上，该研究的亚组分析显示所有的生存获益都只存在于EGFR突变患者，而由于缺乏单用EGFR-TKI的对照组，化疗的加入是否对患者的生存获益有额外贡献不得而知。倒是2015年台湾的一项Ⅱ期临床研究颇值得关注^[8]^，该研究在EGFR野生型患者一线培美曲塞/顺铂方案后随机进行培美曲塞单药维持治疗或培美曲塞联合吉非替尼维持治疗，结果显示培美曲塞联合吉非替尼组PFS显著延长（8.4个月*vs* 3.8个月；HR=0.35；*P*=0.001, 4）。这给我们提供了新的联合治疗思路，但有待大型Ⅲ期临床试验的证实。

对临床实践产生重大影响的当属抗血管生成药物与化疗的联合。2006年发表的ECOG4599研究是晚期NSCLC里程碑式的Ⅲ期临床试验^[9]^，该研究评价了血管内皮细胞生长因子（vascular endothelial growth factor, VEGF）单克隆抗体贝伐珠单抗一线联合紫杉醇/卡铂方案治疗晚期非鳞NSCLC的疗效，结果显示与单纯紫杉醇/卡铂方案化疗相比，可延长约2个月的总生存，首次将晚期NSCLC的生存期延长至了1年以上（12.3个月）。随后另一项Ⅲ期临床试验一线应用贝伐珠单抗与吉西他滨/卡铂方案联合，也得到了相似的阳性结果^[10]^。其后，抗血管生成药物联合化疗的临床试验层出不穷，其中多是抗血管生成的多靶点酪氨酸激酶抑制剂，然而均告失败。直到2014年，一种新的抗血管生成药物Ramucirumab（VEGFR2的人源化IgG1单克隆抗体）在一项Ⅲ期临床试验中与多西他赛联合进行晚期NSCLC的二线化疗^[11]^，与单药多西他赛进行对比，结果OS分别为10.5个月和9.1个月（HR=0.86, 95%CI: 0.75-0.98, *P*=0.023），PFS分别为4.5个月和3.0个月（HR=0.76, 95%CI: 0.68-0.86, *P* < 0.000, 1）。这是近年来在晚期NSCLC二线治疗领域获得的重要进展，尤为值得注意的是该研究纳入了既往被排除于抗血管生成治疗之外的肺鳞癌患者。

近来NSCLC的免疫治疗发展迅猛，尤其是以细胞毒T淋巴细胞相关抗原4（cytotoxic T lymphocyte-associated antigen-4, CTLA-4）单抗和程序性死亡受体1（programmed death 1, PD-1）、细胞程序性死亡配体1（programmed cell death 1 ligand 1, PD-L1）单抗为代表的免疫检查点抑制剂，广义上他们也属于靶向治疗。近期有研究^[12]^报告了一项CTLA4抑制剂伊匹木单抗与化疗联合的Ⅱ期临床试验，NSCLC患者被随机分至3组：安慰剂/化疗组，伊匹木单抗同步联合组（4周期的伊匹木单抗/化疗，随后2周期的安慰剂/化疗），以及伊匹木单抗延迟联合组（2周期安慰剂/化疗，随后4周期伊匹木单抗/化疗）。结果发现延迟联合组可以显著地提高免疫相关无疾病进展生存率，以及在NSCLC患者中可以提高总体无疾病进展生存率。值得注意的是，主要获益的人群是NSCLC患者中的鳞癌患者。

### 2.2 维持化疗

多种化疗药物都曾进行过维持化疗的临床试验，然而或完全失败（如长春瑞滨^[13]^、紫杉醇^[14]^），或仅取得PFS的延长（如多西他赛^[15]^、吉西他滨^[16, 17]^）。而真正取得了维持治疗成功的化疗药物是培美曲塞。2009年发表的JMEN研究^[18]^是一项关于培美曲塞换药维持治疗的多国协作、随机双盲的Ⅲ期临床试验。标准一线含铂双联方案化疗后未进展的晚期NSCLC患者随机接受培美曲塞维持治疗或安慰剂。结果显示PFS（4.3个月*vs* 2.6个月；HR=0.50；*P* < 0.000, 1）和OS（13.4个月*vs* 10.6个月；HR=0.79；*P*=0.012）都获得了有统计学意义的延长。在非鳞癌亚组中，维持治疗组OS的获益更加显著，较对照组延长了5个月之久（15.5个月*vs* 10.3个月；HR=0.70；*P*=0.002）。

不仅如此，培美曲塞在继续维持治疗方面也取得了成功，即Ⅲ期临床试验PARAMOUNT^[19]^。接受4个周期培美曲塞/顺铂方案化疗后的晚期NSCLC，未进展者按2:1随机接受培美曲塞维持治疗或安慰剂。结果显示两组的中位PFS分别为：培美曲赛组3.9个月，安慰剂组2.6个月（*P*=0.000, 06）。维持组和安慰剂组中位OS分别为16.9个月和14.0个月（*P*=0.019, 1），培美曲塞维持治疗降低了22%的死亡风险（HR=0.78）。

这两项临床试验成功地影响了临床实践，使培美曲塞的维持化疗成为临床医生的一种治疗选择。

## 3 化疗疗效预测分子标志物的发展

随着靶向治疗的发展，研究者们开始尝试寻找化疗的疗效预测分子靶标，希望可以筛选出特定化疗药物的获益人群并依此选择化疗药物。

切除修复交叉互补基因1（excision repair cross-complementation group 1, ERCC1）和核糖核苷酸还原酶M1亚基（ribonucleotide reductase M1, RRM1）是这其中研究较早者。ERCC1是修复铂类所导致DNA加合物的重要基因，而RRM1是吉西他滨主要靶点核糖核苷酸还原酶的亚基。因此通常认为ERCC1和RRM1水平分别与铂类、吉西他滨的疗效呈负相关。2006年发表的一项NSCLC术后辅助化疗的大型Ⅲ期临床试验结果显示ERCC1阴性患者接受辅助化疗可延长生存，而阳性者不延长^[20]^。2009年一项Ⅲ期临床试验在晚期NSCLC人群中得到了ERCC1、RRM1与吉西他滨/卡铂化疗的缓解率呈负相关的结果，但与生存期无相关性^[21]^。除此之外，有一项小型临床研究^[22]^曾得出了ERCC1、RRM1水平与相应化疗的近期疗效或生存期呈负相关的结果。但也不乏与之相悖的临床研究。尤其是2013年发表的一项Ⅲ期临床试验^[23]^，入组275例晚期NSCLC患者，2:1随机分组，试验组根据ERCC1和RRM1水平选择化疗方案，分别为：RRM1及ERCC1均低者采用吉西他滨/卡铂方案，RRM1高ERCC1低者采用多西他赛/卡铂方案，RRM1低ERCC1高者采用吉西他滨/多西他赛方案，两者均高者采用多西他赛/长春瑞滨方案。对照组则统一采用吉西他滨/卡铂方案。结果无论ORR、还是PFS和OS，两组间均无统计学差异。但研究者认为本试验结果为假阴性，即Ⅱ类错误，其原因可能为不同中心间标本收集及处理过程的差异，以及所用的检测试剂无法区分ERCC1蛋白的四种亚型——而其中只有一种具有基因修复功能。

研究较多的还有β微管蛋白（β-tubulin），他是抗微管药物（紫杉醇、长春瑞滨等）主要作用位点微管蛋白二聚体的主要组成部分，临床前研究^[24]^发现利用小分子干扰核糖核酸（short interfering RNAs, siRNA）使其亚型TUBB3（β-tubulin Ⅲ）沉默可增加NSCLC细胞系对抗微管药物的敏感性。随后便有小型的临床研究探索TUBB3作为抗微管药物疗效预测因子应用于临床的可能性，但各项研究未得到一致的结论。2007年NSCLC辅助治疗的Ⅲ期临床试验NCIC JB.10进行了探索性分析^[25]^，结果发现术后未接受辅助化疗的患者高TUBB3表达预示着较差的无复发生存期（recurrence free survival, RFS）和OS，接受了辅助化疗的患者高TUBB3表达反而提示RFS和OS延长。2011年一项三药与两药化疗方案对比治疗肺腺癌的Ⅲ期临床研究中评价了TUBB3表达情况与抗微管药物疗效的关系，该研究共入组443例患者，随机接受紫杉醇/顺铂/吉西他滨或顺铂/长春瑞滨方案的化疗，两组共261例患者可评价TUBB3，这些患者中TUBB3阳性者较阴性者PFS（7.87个月*vs* 6.83个月，*P*=0.035）和OS（14.17个月*vs* 11.17个月，*P*=0.018）均有延长。但亚组分析显示获益仅存在于腺癌患者^[26]^。

其他有希望的化疗疗效预测因子还包括与铂类、紫杉类疗效相关的乳腺癌基因1（breast cancer 1, BRCA1）、与培美曲塞相关的胸苷酸合成酶（thymidylate synthase, TS）、与白蛋白结合型紫杉醇相关的富含半胱氨酸的分泌性酸性蛋白（secreted protein acidic and rich in cysteine, SPARC）等，但也均如同上述，缺乏可重复的高级别循证医学证据。因此，目前均不推荐应用于临床。未来需要更多临床研究探索不同研究之间结果相悖的原因，甄别出真正的疗效预测因子。

## 4 小结

综上所述，虽然NSCLC的治疗已经不可避免的跨入了靶向时代，但传统化疗在辅助治疗、局部晚期治疗、解救治疗等方面仍有重要地位，并且随着靶向治疗的发展催生出了新的治疗模式。同时，对于化疗疗效预测靶标的探索仍将继续，使得传统化疗“靶向化”是未来不可避免的发展方向，化疗与靶向治疗的界限将日益模糊。
